# Variability of ^137^Cs and natural radionuclides accumulation in mosses relative to soil activity in Iğdır, Türkiye

**DOI:** 10.3389/fpls.2026.1773571

**Published:** 2026-03-06

**Authors:** Nevzat Batan, Halim Büyükuslu, Nilay Akçay

**Affiliations:** 1 Faculty of Science, Department of Molecular Biology and Genetics, Karadeniz Technical University, Trabzon, Türkiye; 2 Giresun University, Giresun, Türkiye; 3 Faculty of Arts and Sciences, Department of Physics, Recep Tayyip Erdoğan University, Rize, Türkiye

**Keywords:** bioindicator, concentration ratios (CR), gamma ray spectrometry, radioactive accumulation, radiological assessment

## Abstract

**Introduction:**

This study investigates the activity concentrations of ^232^Th, ^226^Ra, ^40^K, and ^137^Cs in soil and moss samples collected from locations in Iğdır Province, Türkiye to evaluate spatial patterns and radionuclide accumulation behavior.

**Methods:**

High-purity germanium (HPGe) gamma spectrometry was used to quantify radionuclide activities.

**Results:**

Statistical analyses included Shapiro–Wilk normality testing, descriptive comparisons between soil and moss, and correlation assessments. Concentration ratios (CR = A_moss_/A_soil_) were calculated to evaluate radionuclide accumulation patterns across species and sites. Spatial variability and multivariate structure were examined using PCA and k-means clustering to identify site- and nuclide-driven grouping patterns. Key radiological parameters calculated for the health risk analysis included absorbed gamma dose rate, internal and external hazard indices, radium equivalent activity, and annual effective dose equivalent. In moss samples, the mean activity concentrations of ^226^Ra, ^232^Th, ^40^K and ^137^Cs were measured as 13.74 ± 0.83 Bq kg^-1^, 13.79 ± 1.1 Bq kg^-1^, 244.72 ± 7.6 Bq kg^-1^, 129.47 ± 1.74 Bq kg^-1^, respectively, and in soil samples, 23.74 ± 0.82 Bq kg^-1^, 22.53 ± 1.11 Bq kg^-1^, 427.01 ± 8.95 Bq kg^-1^, 215.74 ± 1.83 Bq kg^-1^, respectively.

**Discussion:**

All calculated radiological hazard indices, derived from natural radionuclide concentrations, were within permissible recommended limits. Slightly elevated annual effective dose values and absorbed gamma dose rates are observed for the total activity concentrations of both anthropogenic and natural radionuclides, exceeding world population-weighted outdoor averages.

## 1 Introduction

Atmospheric and terrestrial radionuclides enter ecosystems through multiple pathways, including bedrock weathering, soil–water interactions and atmospheric deposition (UNSCEAR, 2000). Among naturally occurring radionuclides, members of the uranium and thorium decay series, as well as ^40^K, constitute the dominant sources of ionizing radiation in most landscapes. Conversely, artificial radionuclides such as ^137^Cs originate primarily from nuclear activities and past fallout events, and are therefore heterogeneously distributed in the environment (Guan et al., 2023). Understanding their environmental behavior is essential because radionuclides exhibit element-specific mobility, retention and bioavailability, and may accumulate in biological compartments through deposition or physiological processes. Soil is of critical importance for the accumulation of environmental pollutants. The pollutants, including heavy metals, pesticides, industrial wastes, and radionuclides, may remain in the soil for extended periods and exert harmful effects on living organisms (Caunii et al., 2015; Ustaoğlu et al., 2024; Yüksel et al., 2024). Factors affecting the activity level of the natural radioactive nuclei ^40^K, ^232^Th, and ^226^Ra in soil include soil type, mineral content, geological activities such as volcanic activity, fault lines and rock composition (Özdemir Öge et al., 2021; Tchorz-Trzeciakiewicz et al., 2023; Wang et al., 2023). High activity concentrations are typically found in regions with certain geological runoff, including as granitic or volcanic rocks, as well as in soils with high clay content (Harb et al., 2012; Saleh et al., 2016; Saleh et al., 2020; Saleh et al., 2021). Regional geological characteristics and groundwater levels are also important mechanisms behind accumulation or dispersion of radioactive materials in the soil. Moreover, mining processes, as a form of human activity, contribute to the release of natural radioisotopes into the water, earth, and atmosphere (Wang et al., 2023; Li et al., 2024).

Unlike vascular plants, mosses lack cuticles and true roots, possess high surface-to-volume ratios, and exchange ions directly with the atmosphere and precipitation through their cell walls (Ding et al., 2024). These characteristics enable them to efficiently intercept airborne particulates, sorb dissolved ions, and store natural and anthropogenic radionuclides in their environment (Butnariu, 2015; Schmidt et al., 2023). Their systems allow direct absorption of water and nutrients from the surrounding air and water (Bates, 2000; Boryło et al., 2017; Antala et al., 2022), with radionuclide transfer occurring through dry deposition and diffusion of aqueous solutions. Mosses act as sensitive biological indicators capable of integrating long-term deposition signals and monitoring ecosystem health, including pollution caused by radionuclides. Spatial variability in radionuclide activity concentrations in mosses can arise from multiple factors, including microtopography, precipitation patterns, canopy cover, substrate contamination and species-specific traits. Species differ in branch density, leaf surface morphology, cation-exchange capacity and growth architecture, all of which modulate interception and retention. As a result, even within the same sampling area, moss species may accumulate markedly different levels of radionuclides.

Their ability to monitor radiation has made mosses the subject of various environmental radiation measurement studies. In Macedonia, the activity levels of Ba, K, La, Rb, Sr, Th, and U in moss samples were measured, and the arithmetic mean of the ambient radiation dose rate was determined to be 110 nSv h^-1^ (Barandovski et al., 2021). Furthermore, in the Marmara region of Türkiye, the activity concentrations of ^137^Cs, ^40^K, ^232^Th, and ^238^U in moss samples ranged from 0.36–8.13, 17.1–181.1, 1.51–6.17, and 0.87–6.70 Bq kg^-1^, respectively (Belivermiş and Çotuk, 2010). Similarly, in Egypt, the activity concentrations of ^238^U, ^226^Ra, ^232^Th, and ^40^K in peat moss samples were reported to range from 17.14–130.83, 13.19–26.09, 5.33–25.2, and 143.26–600.31 Bq kg^-1^, respectively (Elzahraa et al., 2020). In addition, in Fukushima, ^137^Cs activity concentrations in moss samples were found to range from 64 to 105 Bq g^-1^ (Akimoto et al., 2018). On Sobieszewo Island, ^210^Po, ^234^U, and ^238^U were identified in moss samples, with ^210^Po and ^238^U activity concentrations ranging from 133 ± 1 to 501 ± 17 Bq kg^-1^ and from 1.36 ± 0.13 to 3.87 ± 0.10 Bq kg^-1^, respectively, while the ^234^U/^238^U activity ratio was close to unity (0.97 ± 0.05–1.02 ± 0.08) (Boryło et al., 2017). In bryophyte samples collected from the Chornobyl Exclusion Zone, ^137^Cs and ^241^Am activity concentrations were found to reach up to 297 Bq g^-1^ and 0.43 Bq g^-1^, respectively (Schmidt et al., 2023).

Radiation safety involves the measurement and evaluation processes aimed at protecting humans and the environment from radioactive sources (Büyükuslu et al., 2023; Büyükuslu and Akçay, 2026). These measurements are critical for determining radiation levels, monitoring exposure limits, and implementing necessary precautions. Iğdır is important in terms of monitoring radioactivity levels due to its regional environmental sensitivities and proximity to a neighboring country’s nuclear power plant (Metsamor Nuclear Power Plant). Therefore, the region is a suitable study area where natural and potential human-induced effects can be evaluated together. In this study, activity concentrations of ^232^Th, ^226^Ra, ^40^K and ^137^Cs in soil and moss samples were measured, and soil-moss concentration ratios (CR) were calculated to compare radionuclide accumulation between moss and soil. Principal Component Analysis (PCA) and descriptive statistical evaluations were performed to characterize inter-radionuclide associations, identify clustering structures within the dataset. For the soil samples, radiation hazard indicators were estimated to determine potential exposure risks. All radiometric outputs were compared with internationally accepted radiological safety criteria.

## 2 Materials and methods

### 2.1 Study area

In this research, moss and soil samples were collected from various locations within the Iğdır province to assess environmental radioactivity levels. Iğdır is located in a region where high plateaus and mountainous areas cover a large area. Located in the Eastern Anatolia Region, Iğdır has an area of ​​3539 km² and Nakhchivan and Armenia are located to the east. Two-thirds of the surface area of ​​Mount Ağrı, the highest mountain in Türkiye, is within the borders of the Suveren village, a village affiliated to the city center. The surroundings of Mount Büyük Ağrı consist of andesites, and there are young basalt flows that probably erupted from cracks on the slopes of the mountain. Iğdır province and its plain form one of the depressions connected to each other by the Aras river with a number of junctions. The oldest formation in the region is Paleozoic limestones. Iğdır plain is a region with current and intense seismicity due to active faults passing through its immediate surroundings and base (Öztürk, 2023). Iğdır province is quite poor in terms of mineral resources. The only mineral deposit in the province is the rock salt deposit in the Tuzluca field (MTA MT ve AGM). In Iğdır, alluvial, colluvial, chestnut, brown, regosol and basaltic soils have formed. In addition to large soil groups, river floodplains, bare rocks and rubble devoid of soil cover are also seen (Karaoğlu and Çelim, 2018).

**Figure 1:**
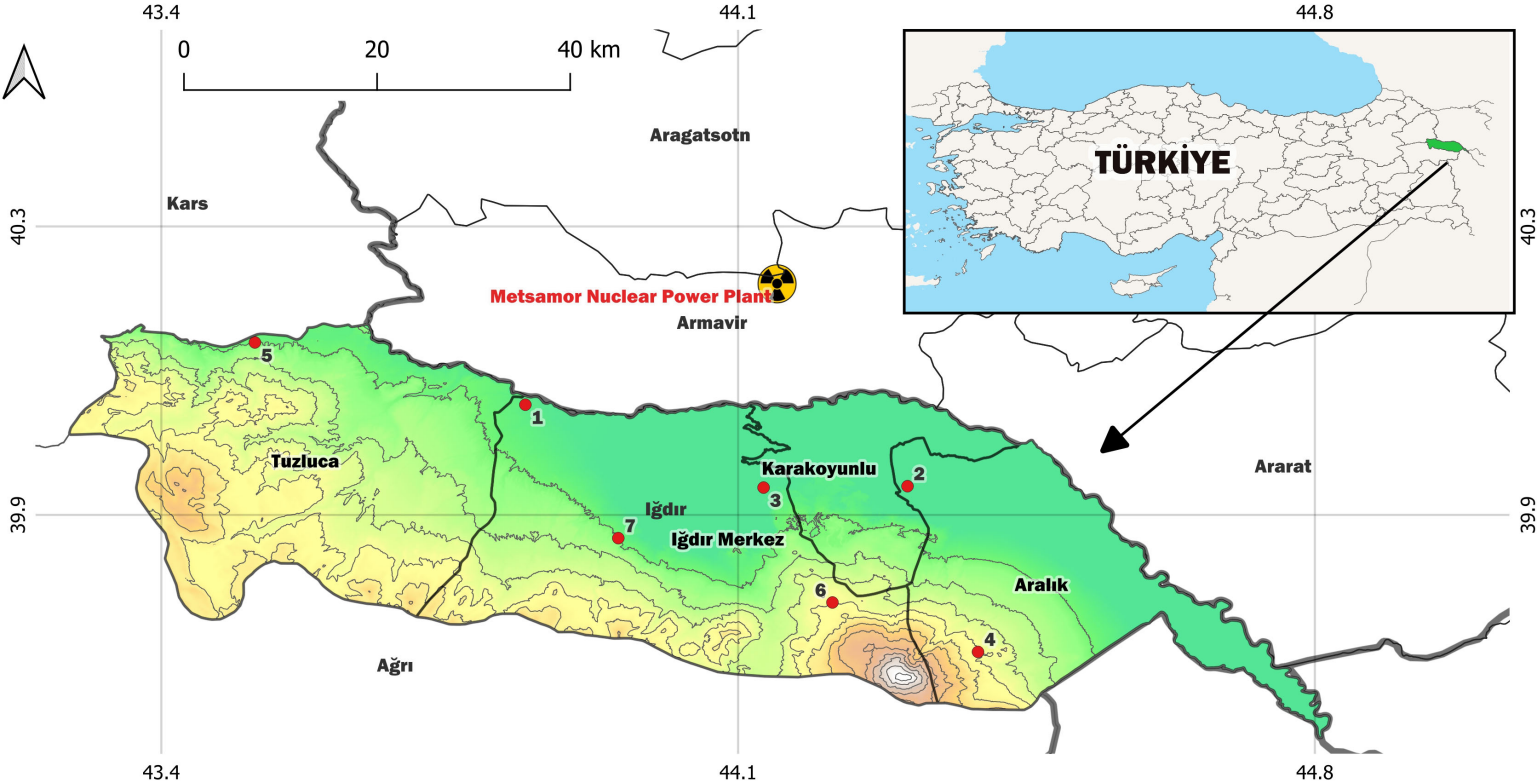
Map of Iğdır Province with the measurement sites and the nuclear power plant.

The Metsamor Nuclear Power Plant (Gürçam, 2022) is situated in the city of Metsamor, Armenia, about 16 kilometers from the Turkish border. The nuclear power plant is located at a distance of 30 km from Iğdır/Türkiye province, which can be considered quite close. The map of Iğdır province, measurement areas and nuclear power plant are picturized in **Figure 1**.

### 2.2 Radioactivity determination in soil and moss samples

#### 2.2.1 Sampling and sample preparation

For radiometric analysis, moss plants and soil samples were gathered from seven different locations near the Armenian border and the interior regions of Iğdır province. In total, seven soil samples and 20 moss plants were obtained from the areas marked in **Figure 1**. Moss samples were collected from multiple locations to represent spatial variability in environmental conditions and potential deposition patterns. At each site, the dominant moss species were sampled from clean patches avoiding mechanical disturbance, runoff channels or direct anthropogenic sources. Soil samples were collected directly beneath or adjacent to the moss layers to ensure spatial consistency between the two sample types. Soils taken from approximately 10 cm below the ground and mass of 1 kg were labeled and filled into bags. The samples were filtered through an 80-mesh sieve (Kırıs, 2022) and placed in an oven at 105 ^0^C (Turhan et al., 2018; Günay et al., 2023; Özden and Aközcan Pehlivanoğlu, 2024) to remove moisture. After staying in the oven for a few days (Kırıs, 2022; Günay et al., 2023; Özden and Aközcan Pehlivanoğlu, 2024), the specimens were situated in polyethylene containers measuring 6 x 5 cm (diameter x height) (100 cm³), configured according to the experimental system, and the containers were plugged and preserved for one month (Turhan et al., 2018; Kırıs, 2022; Özden and Aközcan Pehlivanoğlu, 2024). During this process, the radioactive equilibrium of ^232^Th and ^238^U contained in the specimen was established.

#### 2.2.2 Gamma spectrometric measurements

Gamma spectroscopy was performed using an HPGe detector with appropriate energy calibration, efficiency determination and background subtraction. For the radioactivity measurements of the samples, an Ortec brand GEM55P4–95 Model HPGe detector with a relative efficiency of 55% a resolution of 1.9 keV at 1332.5 keV (Kasumović et al., 2015; Turhan et al., 2018; Elzahraa et al., 2020; Kırıs, 2022) was used. The gamma spectrometer comprises a spectroscopy amplifier, a preamplifier, a detector, and an analog-to- digital converter (ADC) which transforms analog counts into electronic signals, along with a multi-channel analyzer (MCA).

In order to analyze the spectra collected in the multi-channel analyzer, it is necessary to know which channel corresponds to which energy. Each channel corresponds to an energy value. Thus, the types of radioactive nuclei contained in the sample can be found. In order to perform energy calibration, a standard source (s) consisting of nuclei with known energies is needed. With the help of a detector, the spectrum of the standard source for energy calibration was obtained and the channels corresponding to the energies were determined.

Detector efficiency is expressed as the proportion of photons that produce a countable pulse in the detector to the total number of photons arriving at the detector, or as the percentage of photons producing a countable pulse. To assess the accurate magnitude of gamma counts recorded by the detector, it is necessary to apply a correction for detector efficiency. The detector efficiency at the specified energies is calculated using the Equation 1:

(1) $$\epsilon =\frac{s}{(I_{\gamma }).N.t}$$

In this context, t is the counting duration, ϵ signifies the efficiency of the HPGe detector at the respective gamma energy, s corresponds to the net peak area, I_γ_ represents the gamma ray emission probability (Yasmin et al., 2014), and N is the activity of the standard source on the measurement day.

**Figure 2:**
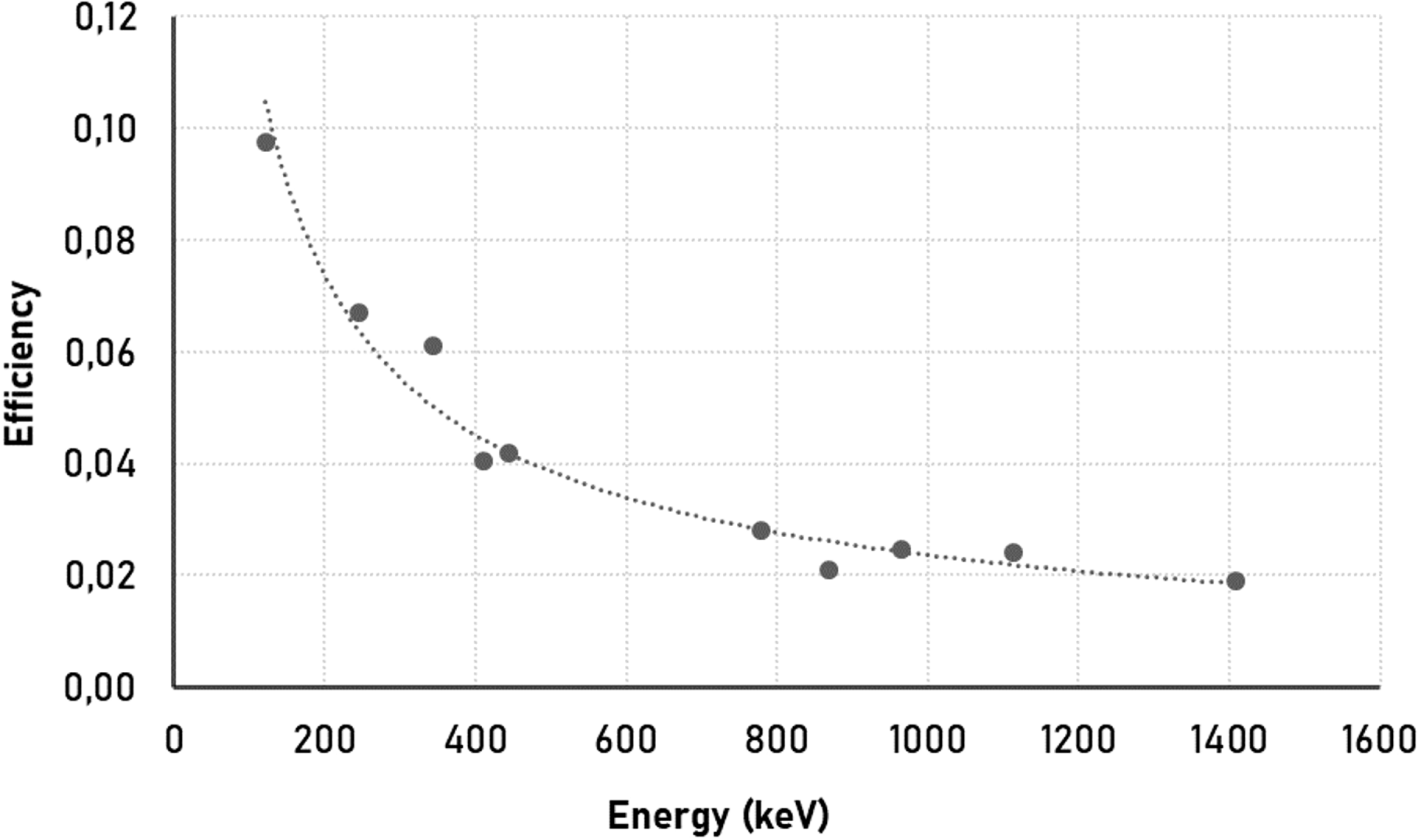
Energy-efficiency calibration curve determined for the detector.

Energy and efficiency calibration of the detector was performed using a liquid ^152^Eu radioactive source. During the calibration process, the characteristic gamma energies of this source, which has different gamma energy levels spanning a wide range, between 121.78 keV and 1408.01 keV, and their corresponding emission probabilities were taken as the basis (IAEA, 2007). **Figure 2** shows the energy-efficiency calibration curve determined for the detector. After a one-month waiting period to ensure radioactive balance, measurements were started. The supply voltage of the HPGe detector used in the counts was 4000 V. Genie-2000 was used as the counting programme. The sample counting period was selected as 50000 s. At the end of this period, the spectra of the radioactive isotopes emitted from the samples were obtained.

For each peak in the spectrum, the relevant areas were identified, and the peak area was accurately delineated to obtain the net area with minimal error. The energy levels identified at the center channel of each peak were determined using calibration factors and a computer program. These steps ensured precise and reliable analysis of the spectral data.

To calculate activity concentrations, the known energies and emission rates of specific radionuclides were utilized. For the ^238^U (^226^Ra) series, the peak areas corresponding to ^214^Pb (295.2 keV and 352.0 keV) and ^214^Bi (609.4 keV) (Kalkan et al., 2020) were analyzed. Similarly, for the ^232^Th series, the peak areas at ^208^Tl (583.1 keV), ^228^Ac (911.1 keV), and ^212^Pb (238.6 keV) (Nguelem et al., 2017) were used. For ^40^K, the peak area at 1460.8 keV was employed in the calculations. These precise measurements ensured accurate determination of the radionuclide activities.

Accurate activity calculations require isolating net peak areas from the measured spectrum. The net areas under the peaks are derived from the total area by deducting the background counts. Counts per second (cps) is the unit representing the area under the peak divided by the duration required to achieve the peak. Considering detector efficiency, the activity is as in Equation 2:

(2) $$A=\frac{s}{(I_{\gamma }).w.t.\epsilon }$$

where A (Bq kg^-1^) represents activity, s is a net area, $I_{\gamma }$ is a gamma ray emission rate, *w* (kg) is the sample mass and *ϵ* is an efficiency.

### 2.3 Concentration ratio

Radionuclide accumulation in mosses is commonly expressed using the concentration ratio (CR), defined as the ratio of radionuclide activity concentration in moss to that in the adjacent soil. To quantify radionuclide accumulation, the concentration ratio (CR) was defined as in Equation 3 (Dowdall et al., 2005; Zhong et al., 2019; Guan et al., 2023):

(3) $$CR=\frac{moss\;activity\;\;concentration\;(Bq\cdot kg^{-1}dry\;weight)}{soil\;activity\;conccentration\;(Bq\cdot kg^{-1}dry\;weight)}$$

CR values represent the relative accumulation potential of moss species and are not equivalent to true soil-to-plant transfer indices. This ratio does not reflect physiological soil-to-plant transfer but integrates both atmospheric deposition and surface sorption mechanisms inherent to mosses. Therefore, CR was interpreted as a relative accumulation index rather than an uptake factor.

### 2.4 Radiological hazard assessments for soil samples

Radiological hazard indices provide quantitative measures to evaluate the potential risks associated with natural and anthropogenic radionuclides in soil samples.

Radium equivalent activity (Ra_eq_), as give in Equation 6, is a comprehensive activity criterion utilized to assess the individual activity concentration of materials with varying concentrations of ^232^Th, ^40^K, and ^226^Ra (Beretka and Mathew, 1985; Isinkaye and Emelue, 2015). Two of the indicators that determine the radiation hazard criteria of materials are external and internal hazard indexes (Equations 4 and 5). $\;H_{ex}$ only considers external hazards from γ-rays, while $H_{in}$ considers internal hazards the effects of Radon gas and also its by-products (Beretka and Mathew, 1985). Natural and anthropogenic radionuclide gamma dose rate determined (Equations 7 and 8) at a height of 1 meter above earth level (UNSCEAR, 2000) is one of the indicators used as the basis for radiation hazard assessment. United Nations Scientific Committee on the Effects of Atomic Radiations (UNSCEAR) (UNSCEAR, 2000) supplied the equation for the ^226^Ra ^232^Th, and ^40^K radionuclides. The annual effective dose equivalent (AEDE) of communities affected by radioactivity is related to the gamma dose rate. To estimate the (AEDE) for individuals exposed to gamma radiation in Equation 9, the gamma dose rate is considered in conjunction with a 0.7 Sv Gy^-1^ conversion factor for humans and occupancy factors of 0.2 for outdoor exposure and 0.8 for indoor exposure (UNSCEAR, 2000).

In this study, radiological hazard indices—including radium equivalent activity (Ra_eq_), external (H_ex_) and internal (H_in_) hazard indices, absorbed gamma dose rate (D_out_), and the annual effective dose equivalent (AEDE)—were calculated for the soil samples, and the formulas used are presented in **Table 1**.

**Table 1:** Equations and threshold values for radiological hazard indices.

Index	Formula	Limit/world mean
$H_{ex}$	(4) $$\left(\frac{C_{Ra}}{370BqKg^{-1}})+(\frac{C_{Th}}{259BqKg^{-1}})+(\frac{C_{K}}{4810BqKg^{-1}}\right)$$	≤ 1
$H_{in}$	(5) $$\left(\frac{C_{Ra}}{185BqKg^{-1}})+(\frac{C_{Th}}{259BqKg^{-1}})+(\frac{C_{K}}{4810BqKg^{-1}}\right)$$	≤ 1
$Ra_{eq}$	(6) $$C_{Ra}+1.43C_{Th}+0.07C_{K}$$	370 Bq kg^-1^
$D(nGyh^{-1})$	(7) $$0.462C_{Ra}+0.604C_{Th}+0.0417C_{K}$$	≈ 59 nGy h^-1^
	(8) $$0.462C_{Ra}+0.604C_{Th}+0.0417C_{K}+0.1243C_{Cs}$$	
$AEDE_{out}(mSvy^{-1})$	(9) $$D(nGyh^{-1})\times 8760hy^{-1}\times 0.7\times 10^{-6}\times 0.2$$	≤ 0.07 mSv y^-1^

In the equations listed in **Table 1**, $C_{K}$, $C_{Th}$, $C_{Cs}\;$and $C_{Ra}$, correspond to the activity concentrations of ^40^K, ^232^Th, ^137^Cs, and ^226^Ra respectively (ICRP, 1990; Isinkaye and Emelue, 2015).

Total absorbed dose rates are a quantity related to all radioactive nuclei in the soil content. When considering the contribution of the anthropogenic radionuclide ^137^Cs, the equation for the total absorbed dose rate is adjusted in Equation 8 (Clouvas et al., 2000; Rafique et al., 2013).

### 2.5 Statistical analysis

Descriptive statistics were used to evaluate the average and variability of radionuclide concentrations. Considering the sample size, normality was assessed using the Shapiro–Wilk test. Principal component analysis (PCA) was conducted to identify clustering patterns among radionuclide groups and moss species, focusing on variations in environmental conditions rather than on causal relationships.

## 3 Result and discussion

### 3.1 Activity levels of ^40^K, ^137^Cs ^226^Ra, and ^232^Th

**Table 2** listed details of the moss and soil samples, including moss sample names, sample numbers, collection sites, altitudes, and geographic coordinates. It also lists the measured activity concentrations of ^40^K, ^137^Cs, ^226^Ra, and ^232^Th for each sample. In soil samples, the activity levels of ^226^Ra vary between 18.23 ± 0.77 and 27.75 ± 0.86 Bq kg^-1^, with a mean magnitude of 23.74 ± 0.82 Bq kg^-1^. The ^232^Th activity levels vary between 19.30 ± 1.08 and 24.45 ± 1.14 Bq kg^-1^, with a mean of 22.53 ± 1.11 Bq kg^-1^. For the radionuclide ^40^K, activity concentrations range from 398.02 ± 9.07 to 463.46 ± 8.54 Bq kg^-1^, with a mean magnitude of 427.01 ± 8.95 Bq kg^-1^. In addition, the anthropogenic radionuclide ^137^Cs exhibits activity concentrations in soil samples ranging from 52.71 ± 0.98 to 419.49 ± 2.68 Bq kg^-1^, with a mean value of 215.74 ± 1.83 Bq kg^-1^.

**Table 2:** Overview of the study area, descriptive information of moss and soil samples, and their measured ^232^Th, ^226^Ra, ^137^Cs and ^40^K activity concentrations.

1. Tuzluca (933m): 40.033611, 43.841667					
Sample	No	^232^Th (Bq kg^-1^)	^226^Ra (Bq kg^-1^)	^137^Cs (Bq kg^-1^)	^40^K (Bq kg^-1^)
*Syntrichia ruralis*	M11	19.88 ± 1.29	17.8 ± 0.93	127.86 ± 1.87	284.3 ± 0.02
*Hypnum cupressiforme*	M12	19.57 ± 2.38	27.48 ± 1.97	200.54 ± 3.65	348.8 ± 16.04
*Homalothecium sericecum*	M13	16.24 ± 0.92	13.57 ± 0.68	210.34 ± 1.85	255.02 ± 6.78
Soil	S1	24.45 ± 1.14	27.75 ± 0.86	153.88 ± 1.63	419.55 ± 8.87
2. Aralık (832m): 39.934722, 44.305556					
Sample	No	^232^Th (Bq kg^-1^)	^226^Ra (Bq kg^-1^)	^137^Cs (Bq kg^-1^)	^40^K (Bq kg^-1^)
*Grimmia laevigata*	M21	9.41 ± 1.08	8.01 ± 0.92	156.19 ± 2.2	235.74 ± 8.79
Soil	S2	19.3 ± 1.08	18.23 ± 0.77	127.3 ± 1.51	425.38 ± 9.15
3. Karakoyunlu (Melekli) (858m): 39.933056, 44.130833					
Sample	No	^232^Th (Bq kg^-1^)	^226^Ra (Bq kg^-1^)	^137^Cs (Bq kg^-1^)	^40^K (Bq kg^-1^)
*Grimmia ovalis*	M31	14.3 ± 0.82	12.96 ± 0.65	208.07 ± 1.73	236.79 ± 6.31
*Grimmia laevigata*	M32	14.94 ± 1.12	16.16 ± 0.81	168.76 ± 1.91	244.78 ± 7.71
*Syntrichia ruralis*	M33	12.74 ± 1.4	5.89 ± 1.1	132.34 ± 2.44	165.55 ± 8.78
Soil	S3	21.93 ± 1.04	20.24 ± 0.73	52.71 ± 0.98	402.75 ± 8.56
4. Aralık (Yenidogan Village) (2086m): 39.733611, 44.391111					
Sample	No	^232^Th (Bq kg^-1^)	^226^Ra (Bq kg^-1^)	^137^Cs (Bq kg^-1^)	^40^K (Bq kg^-1^)
*Hypnum cupressiforme*	M41	13.91 ± 1.07	19.42 ± 0.75	27.56 ± 0.75	277.58 ± 7.57
*Homalothecium lustecens*	M42	12.71 ± 0.95	17.01 ± 0.78	50.11 ± 1.1	240.17 ± 7.61
*Grimmia ovalis*	M43	15.13 ± 1.11	17.73 ± 0.89	233.37 ± 2.38	301.01 ± 9.02
Soil	S4	22.17 ± 1.13	26.68 ± 0.94	385.47 ± 2.54	437.18 ± 9.23
5. Tuzluca and Kagızman (1049m): 40.109167, 43.513333					
Sample	No	^232^Th (Bq kg^-1^)	^226^Ra (Bq kg^-1^)	^137^Cs (Bq kg^-1^)	^40^K (Bq kg^-1^)
*Syntrichia Ruralis*	M51	14.82 ± 0.99	18.02 ± 0.81	141.13 ± 1.72	228.89 ± 7.28
*Grimmia longirostiris*	M52	18.19 ± 1.89	11.62 ± 1.23	123.39 ± 2.5	325.95 ± 13.22
*Syntrichia virecens*	M53	12.45 ± 0.67	13.23 ± 0.5	101.51 ± 1.09	238.28 ± 5.58
Soil	S5	24.31 ± 1.24	25.5 ± 0.9	419.49 ± 2.68	442.71 ± 9.26
6. Korhan (Agrı mount) (2088m): 39.793611, 44.214444					
Sample	No	^232^Th (Bq kg^-1^)	^226^Ra (Bq kg^-1^)	^137^Cs (Bq kg^-1^)	^40^K (Bq kg^-1^)
*Hypnum cupressiforme*	M61	11.97 ± 0.82	12.68 ± 0.6	24.04 ± 0.7	271.14 ± 7.22
*Syntrichia ruralis*	M62	12.8 ± 0.74	15.97 ± 0.61	132.51 ± 1.3	221.52 ± 5.54
*Grimmia ovalis*	M63	15.53 ± 0.8	18.18 ± 0.61	184.71 ± 1.5	253.19 ± 5.9
*Homalothecium sericecum*	M64	10.85 ± 1.23	7.22 ± 0.82	105.01 ± 1.89	197.25 ± 8.52
Soil	S6	24.06 ± 1.17	22.65 ± 0.83	219.09 ± 2.02	398.02 ± 9.07
7. Halfeli (1077m): 39.871667, 43.954444					
Sample	No	^232^Th (Bq kg^-1^)	^226^Ra (Bq kg^-1^)	^137^Cs (Bq kg^-1^)	^40^K (Bq kg^-1^)
*Grimmia laevigata*	M71	9.8 ± 0.85	8.11 ± 0.67	123.82 ± 1.67	184.66 ± 6.62
*Grimmia longirostris*	M72	10.85 ± 0.86	10.73 ± 0.67	78.88 ± 1.28	216.14 ± 6.73
*Homalothecium sericecum*	M73	9.63 ± 0.93	3.02 ± 0.63	59.19 ± 1.31	167.58 ± 6.83
Soil	S7	21.49 ± 0.99	25.15 ± 0.74	152.25 ± 1.45	463.46 ± 8.54

In moss samples, the activity levels of ^226^Ra varied between 3.02 ± 0.63 (*Homalothecium sericecum*) and 27.48 ± 1.97 (*Hypnum cupressiforme*) Bq kg^-1^, with an arithmetic mean of 13.74 ± 0.83 Bq kg^-1^. The measured ^232^Th activity concentrations varied between 9.41 ± 1.08 (*Grimmia laevigata*) and 19.88 ± 1.29 (*Syntrichia ruralis*) Bq kg^-1^, with a mean of 13.79 ± 1.10 Bq kg^-1^. Similarly, the activity concentrations of ^40^K ranged from 165.55 ± 8.78 (*Syntrichia ruralis*) to 348.80 ± 16.04 (*Hypnum cupressiforme*) Bq kg^-1^, with a mean value of 244.72 ± 7.60 Bq kg^-1^. For ^137^Cs, the activity concentrations ranged from 24.04 ± 0.70 (*Hypnum cupressiforme*) to 233.37 ± 2.38 (*Grimmia ovalis*) Bq kg^-1^, with an arithmetic mean of 129.47 ± 1.74 Bq kg^-1^ for all moss samples.

The activity concentrations of ^226^Ra and ^232^Th in the moss samples showed similar values, and a significant linear correlation of R^2^ = 0.52 was obtained between these two radionuclides. A similar relationship was observed in soil samples, with a linear correlation of R^2^ = 0.45 calculated for ^226^Ra and ^232^Th. In addition, it is noteworthy that the ^137^Cs activity concentrations measured in the soil at locations 4, 5, and 6 were higher than at other locations. Altitude, one of the factors affecting atmospheric accumulation, may explain this situation, as these locations represent the highest altitude areas in the study area. In particular, the ^137^Cs activity concentration in moss at locations 1, 2, and 3 is observed to be higher than the values in the soil; because moss can retain ^137^Cs accumulation for a long time due to their large surface areas, strong ion exchange and chelation capacities, and thick and widespread cover, thus acting as a biological barrier in the ecosystem by slowing the transfer of radioactive cesium to the soil.

The activity level ratio magnitude of soil samples compared to moss samples were 57.3% for ^40^K, 57.8% for ^226^Ra, 60% for ^137^Cs, and 61.2% for ^232^Th, respectively. The trend observed in the arithmetic averages of radionuclide concentrations is: ^232^Th ≤ ^226^Ra <^137^Cs <^40^K. According to global population-weighted averages, the radionuclide activity level in soil samples are 32 Bq kg^-1^ for ^226^Ra, 45 Bq kg^-1^ for ^232^Th, and 420 Bq kg^-1^ for ^40^K (UNSCEAR, 2000). The quantified levels of ^226^Ra and ^232^Th in this study were below the world population-weighted averages, whereas the level of ^40^K was slightly above. The ^137^Cs activity concentration can be compared with findings from similar studies within the country, as referenced (Taskin et al., 2009; Celik et al., 2010; Ozaydin Ozkara et al., 2021; Günay et al., 2023; Özden and Aközcan Pehlivanoğlu, 2024), it is seen that the activity concentration in soil samples values ​​in this study are higher.

A significant inter-species difference in radioactivity accumulation was observed in moss samples. *Grimmia* species, particularly *G. ovalis* and *G. laevigata*, exhibited the highest ^137^Cs concentrations in most study areas. This trend may be consistent with the possible effects of surface morphology and tissue differences in some mosses on radionuclide accumulation. In contrast, *Homalothecium* species were represented by lower activity levels for both natural radionuclides and ^137^Cs. *Syntrichia* species, on the other hand, formed an intermediate group, showing a moderate and relatively balanced accumulation pattern for all isotopes. The average activity concentrations measured in epigeic moss *H. cupressiforme* were determined as 15.15 for ^232^Th, 299.17 for ^40^K, and 84.04 for ^137^Cs. These values are considerably higher than the ranges reported by Belivermiş and Çotuk (2010) (1.51–6.17 for ^232^Th, 17.1–181.1 for ^40^K, and 0.36–8.13 for ^137^Cs) in their study conducted in the Marmara Region of Türkiye. Similarly, S. Dragović and et al (Dragović et al., 2010), also detected the highest activity concentrations in the species *H. cupressiforme*. Differences in radioactivity accumulation among moss species stem from species-specific physiological and physical characteristics such as surface morphology, growth strategies, and particle retention capacity. Additionally, ecological interactions between bryophytes and other plants, as well as adsorption levels, contribute to these differences (Ren et al., 2021). It has been emphasized that radionuclide uptake by moss depends on the ecological conditions (altitude, precipitation rate) at the place of growth (Nifontova, 2000; Petrinec et al., 2022).

**Figure 3:**
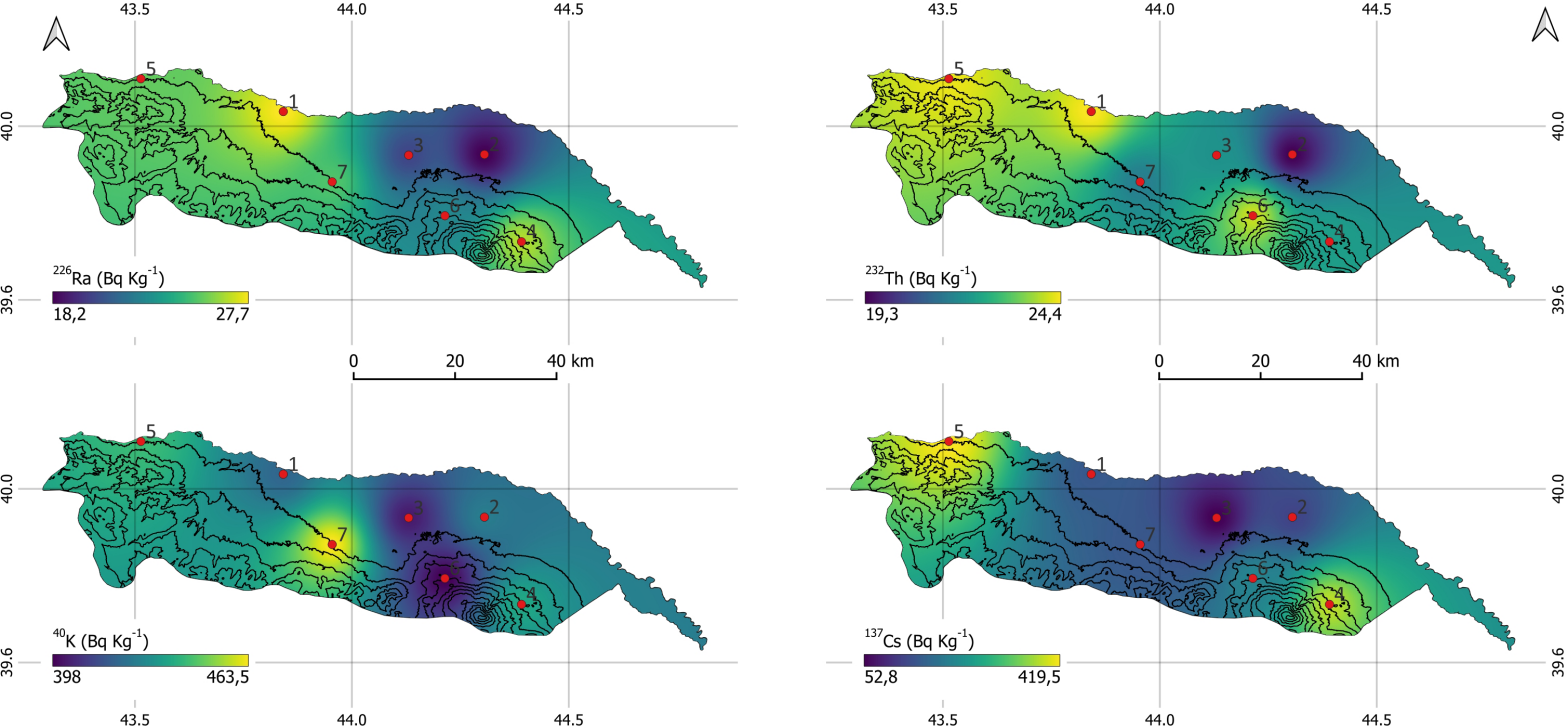
Spatial distribution maps of ^232^Th, ^226^Ra, ^40^K and ^137^Cs radioactivity concentrations in the soil samples collected from the Iğdır province.

The spatial distribution maps of radioactivity concentrations in soil samples from the Iğdır province are presented in **Figure 3**. The spatial distribution maps of ^232^Th and ^226^Ra show that both radionuclides exhibit a similar pattern, with high values concentrated in the west–northwest around S1 and S5, and low values concentrated in the eastern section around S2 and S3. This overlap reveals a clear spatial correlation between the two members of the natural series. In contrast, ^40^K shows a different distribution, with high values concentrated more in the central-southern belt, particularly around points S4 and S7. ^137^Cs, on the other hand, diverges from the natural radionuclides, presenting a more heterogeneous and localized pattern; the highest values are observed around S4 and S5, deviating from the more regular distributions seen in the other maps.

Descriptive statistics of the measured and ^137^Cs, ^40^K, ^232^Th, and ^226^Ra, activity levels are listed in **Table 3**. The descriptive statistics of ^232^Th, ^226^Ra, ^40^K and ^137^Cs radionuclides in soil and moss samples from Iğdır province indicate distinct patterns between the two sample types. For natural radionuclides, soil samples consistently exhibit higher arithmetic mean, median, and maximum values compared to moss samples, reflecting a generally higher radionuclide content in the soil samples. In contrast, moss samples show greater relative variability for some radionuclides, as evidenced by higher standard deviations, particularly for ^40^K. Skewness values reveal that ^232^Th and ^226^Ra in soils are slightly negatively skewed, whereas moss values are closer to symmetric distributions. ^137^Cs exhibits noticeable heterogeneity, with strongly positive skewness in soil and near-symmetric distribution in moss, highlighting localized accumulation. All kurtosis and skewness values remain within the range of (–1, 1). Shapiro–Wilk test results (p > 0.05) suggest that most radionuclide datasets do not significantly deviate from normality. Overall, these statistics underscore the consistent enrichment of natural radionuclides in soil, contrasted with more heterogeneous and variable patterns in moss, while ^137^Cs displays a markedly irregular distribution across both sample types.

**Table 3:** Descriptive statistics.

*Statistic*	^232^Th (Bq kg^-1^)		^226^Ra (Bq kg^-1^)		^40^K (Bq kg^-1^)		^137^Cs (Bq kg^-1^)	
	*Soil*	*Moss*	*Soil*	*Moss*	*Soil*	*Moss*	*Soil*	*Moss*
*min.*	19.30	9.41	18.23	3.02	398.02	165.55	52.71	24.04
*max.*	24.45	19.88	27.75	27.48	463.46	348.8	419.49	233.37
*arithmetic mean*	22.53	13.79	23.74	13.74	427.01	244.72	215.74	129.47
*geometric mean*	22.46	13.47	23.51	12.40	426.48	240.22	177.58	110.93
*median*	22.17	13.36	25.15	13.40	425.38	239.23	153.88	130.10
*standard deviation*	1.88	3.09	3.50	5.68	22.97	48.13	136.99	61.03
*skewness*	-0.66	0.49	-0.62	0.23	0.28	0.34	0.71	-0.13
*kurtosis*	-0.19	-0.36	-0.99	0.57	-0.55	0.08	-0.96	-0.74
*Shapiro–WilkTest (p)*	0.321	0.347	0.574	0.665	0.888	0.796	0.265	0.664

**Figure 4:**
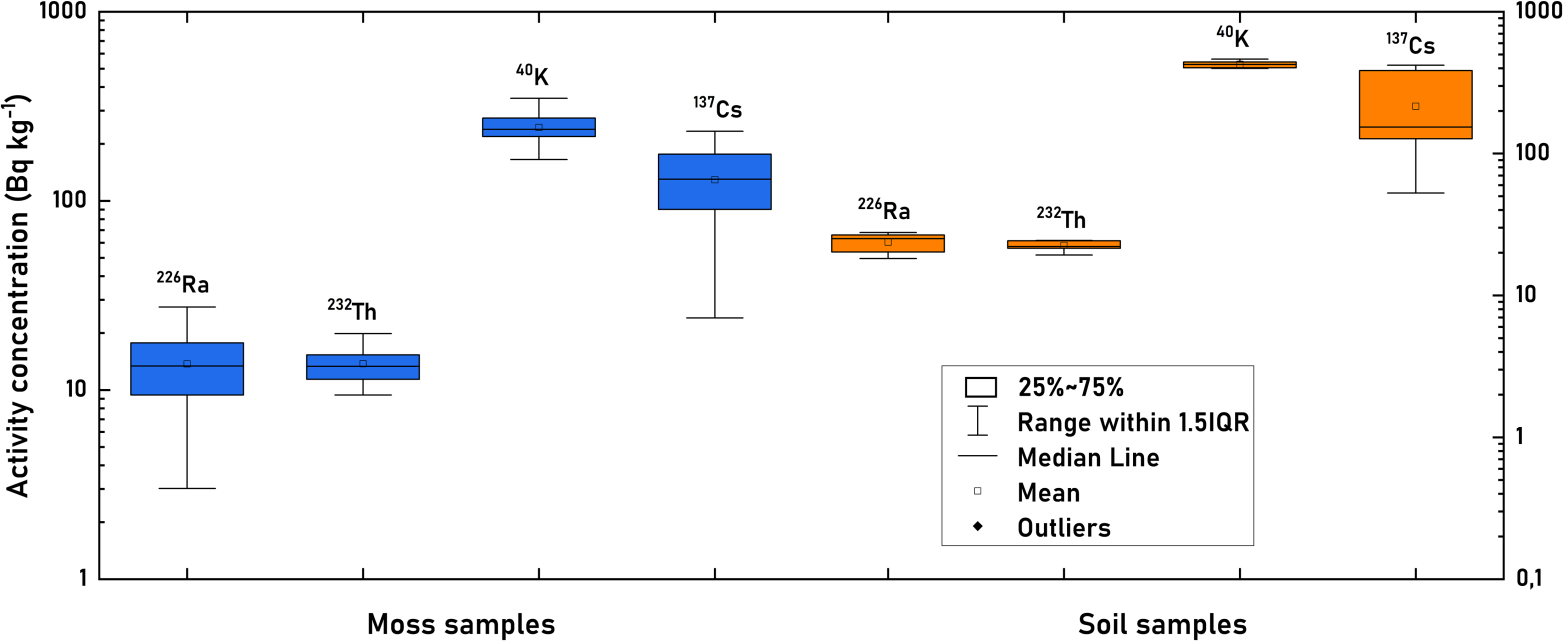
Box plots of the ^226^Ra, ^232^Th, ^40^K, ^137^Cs, and radionuclides in the moss and soil subjects.

Box plots, shown in **Figure 4**, provide a clearer visualization of the data distribution for soil and moss subjects. The activity levels of ^232^Th and ^226^Ra demonstrate a narrow range and low variance, reflecting their consistently low levels in both sample types. Conversely, ^40^K and ^137^Cs exhibit greater variability and higher activity concentrations, with ^137^Cs, in particular, showing a wide data spread in soil samples, especially at the lower values.

Regional activity concentration measurements including surrounding provinces and activity concentrations for Iğdır were made by various researchers (Topcuoğlu et al., 2003; Turhan et al., 2018; Karahan et al., 2020). The results of activity concentration measurements from previous studies on soil samples, along with the findings of the current research, are displayed in **Table 4**.

**Table 4:** Overview of ^40^K, ^137^Cs, ^226^Ra, and ^232^Th activity levels in soil subjects from Iğdır province compared to those from other countries.

Region	^232^Th (Bq kg^-1^)	^226^Ra (Bq kg^-1^)	^40^K (Bq kg^-1^)	^137^Cs (Bq kg^-1^)	Ref.
*Iğdır*	14.1 ± 5.2	13 ± 12.6	441 ± 182	16 ± 6.0	(Karahan et al., 2020)
	21.9	19.1	437.7	11.8	(Turhan et al., 2018)
	5 ± 1	25 ± 9 (^238^U)	352 ± 23	13 ± 1	(Topcuoğlu et al., 2003)
*İstanbul (Türkiye)*	–	–	–	< 0.07 – 9.95	(Özden et al., 2024)
	30 ± 2	26 ± 2	540 ± 29	0.55 ± 0.07	(Günay et al., 2024a)
*Konya (Türkiye)*	–	–	–	0.74 – 12.88	(Günay et al., 2023)
	10.19 – 46.09	14.07 – 67.27	107.87 – 605.95	–	(Özden et al., 2023)
*Salda Lake (Türkiye)*	3.19	2.68	270.80	3.41	(Günay et al., 2025)
*Kocaeli (Türkiye)*	26.36	22.35 (^238^U)	368.34	2.44	(Günay et al., 2024b)
*This study*	22.53 ± 1.11	23.74 ± 0.82	427.01 ± 8.95	215.74 ± 1.83	
*Türkiye (arithmetic mean)*	33.0 ± 0.7	28.6 ± 0.7 (*^238^U*)	448.5 ± 7.3	13.4 ± 0.8	(Turhan et al., 2012)
*Yemen (Aden Assoghra)*	22.1 ± 0.64	13 ± 0.44	509 ± 9	0.3 – 1.7	(Abdullah and Saleh, 2023)
*Yemen (Assalamia-Alhomira)*	211.5 ± 14	80.77 ± 4.5	1004.8 ± 40	–	(El-Kamel et al., 2012)
*Spain (Eastern Canary Islands)*	28.9	25.2	384.4	–	(Arnedo et al., 2017)
*Southern China (Guangdong)*	101.0	75.1	535.8	–	(Yang and Sun, 2022)
*Bosnia and Herzegovina (Tuzla and Lukavac)*	32	32	331	37	(Kasumović et al., 2015)
*North Cyprus*	53.6±5.9	83.7±9.0	593.9±54.0	7.1±0.8	(Abbasi et al., 2020)
*India (Dhanbad)*	64.5	60.3	481.0	–	(Zubair and Shafiqullah, 2020)
*World (median)*	30	35	400	–	(UNSCEAR, 2000)

A key observation from the comparison is the remarkably higher ^137^Cs activity concentration found in this study compared to other measurements. This result may be related to various factors such as climate characteristics, differences in measurement dates, distribution of measurement areas, and activity status of nearby nuclear power plants. While the results of ^232^Th are more compatible with the results by Turhan et al (Turhan et al., 2018),, the results by Karahan et al. (2020), and Topcuoğlu et al. (2003) have lower values. According to the comparison of ^226^Ra activity concentration results, measurements other than (Karahan et al., 2020) have almost close values. ^40^K activity concentrations had values ​​close to each other in the range of 427–441 Bq kg^-1^. All mean of radionuclide activity level measurement results, except for ^137^Cs are below the Türkiye arithmetic means (Turhan et al., 2012) of natural and human-induced radionuclide activity levels listed in **Table 3**. Moreover, there is insufficient literature available to compare natural radionuclides and ^137^Cs activity concentrations in the water sources of Iğdır province; only a study by Gülay et al. (2018) (2013–2015) examined ^137^Cs levels in drinking water, reporting concentrations from three different locations ranging between <0.67 and <2.80 Bq/m^3^.

### 3.2 Concentration ratio

The concentration ratios (CR) obtained in this study reinforce the role of mosses as effective indicators of environmental deposition rather than as physiological uptake systems. Since mosses lack functional roots, nutrient-conducting tissues and cuticular barriers, their interaction with radionuclides is driven by passive interception, ion exchange and surface adsorption. Consequently, CR values reflect a combined signature of atmospheric inputs, particulate trapping and substrate contamination instead of directional transfer from soil. The elevated CR values of fallout-derived radionuclides compared to natural radionuclides therefore should be interpreted as evidence of persistent deposition and prolonged retention in moss biomass, rather than enhanced biological absorption. In this study, the CR values calculated from the radioactivity concentrations measured in the moss and soil samples are listed in **Table 5**.

**Table 5:** CR values calculated from the radioactivity concentrations measured in moss and soil samples.

Moss sample	Soil sample	CR (^232^Th)	CR (^226^Ra)	CR (^137^Cs)	CR (^40^K)
*Syntrichia ruralis*	S1	0.81	0.64	0.83	0.68
*Hypnum cupressiforme*	S1	0.80	0.99	1.30	0.83
*Homalothecium sericecum*	S1	0.66	0.49	1.37	0.61
*Grimmia laevigata*	S2	0.49	0.44	1.23	0.55
*Grimmia ovalis*	S3	0.65	0.64	3.95	0.59
*Grimmia laevigata*	S3	0.68	0.80	3.20	0.61
*Syntrichia ruralis*	S3	0.58	0.29	2.51	0.41
*Hypnum cupressiforme*	S4	0.63	0.73	0.07	0.63
*Homalothecium lustecens*	S4	0.57	0.64	0.13	0.55
*Grimmia ovalis*	S4	0.68	0.66	0.61	0.69
*Syntrichia Ruralis*	S5	0.61	0.71	0.34	0.52
*Grimmia longirostiris*	S5	0.75	0.46	0.29	0.74
*Syntrichia virecens*	S5	0.51	0.52	0.24	0.54
*Hypnum cupressiforme*	S6	0.50	0.56	0.11	0.68
*Syntrichia ruralis*	S6	0.53	0.71	0.60	0.56
*Grimmia ovalis*	S6	0.65	0.80	0.84	0.64
*Homalothecium sericecum*	S6	0.45	0.32	0.48	0.50
*Grimmia laevigata*	S7	0.46	0.32	0.81	0.40
*Grimmia longirostris*	S7	0.50	0.43	0.52	0.47
*Homalothecium sericecum*	S7	0.45	0.12	0.39	0.36

CR values varied substantially across moss species and sampling sites. Natural radionuclides typically yielded CR<1, consistent with soil-bound dominance and limited particulate transport. Conversely, fallout-derived radionuclides, particularly ^137^Cs, commonly produced CR>1, highlighting the effectiveness of mosses as long-term atmospheric collectors. The calculated concentration ratios for ^232^Th and ^226^Ra mostly ranged between 0.4 and 0.8, indicating that moss species cannot accumulate these natural radioisotopes as effectively as soil. The highest CR values were calculated as 0.81 for ^232^Th in the *Syntrichia ruralis*–S1 soil pair and 0.99 for ^226^Ra in the *Hypnum cupressiforme*–S1 soil pair. In contrast, the lowest CR values were calculated as 0.12 for ^226^Ra and 0.45 for ^232^Th in *Homalothecium sericeum*–S6 (S7) soil samples, respectively. The results reveal that both species-level biological differences and sampling site-specific environmental conditions have a significant effect on radioisotope bioaccumulation. The CR values for ^137^Cs, however, vary over a much wider range (0.07–3.95), indicating that ^137^Cs accumulation is strongly influenced by both species-specific characteristics and regional conditions. The highest CR was calculated as 3.95 in the *Grimmia ovalis*–S3 soil pair, indicating that this species accumulates approximately four times more ^137^Cs from the soil. In contrast, the CR value was calculated to be 0.07 in *Hypnum cupressiforme*–S4 soil samples, indicating that this species showed almost no accumulation. In this study area and under these environmental conditions, certain *Grimmia* species in particular were found to have a high biological accumulation potential for ^137^Cs. The CR values calculated for ^40^K are concentrated in the range of 0.36–0.74, indicating that moss accumulate potassium from the soil at a moderate level. The fact that CR remains in the range of 0.36–0.55 in *Homalothecium* species reveals that this group has a relatively lower bioaccumulation capacity for ^40^K.

The average CR values obtained in this study for ^232^Th, ^226^Ra, ^137^Cs, and ^40^K (geom.: 0.59; 0.52; 0.60; 0.57; arith.: 0.60; 0.56; 0.99; 0.58) are generally consistent with values reported in the literature. In Serbia (Dragović et al., 2010), ^137^Cs (2.39) is higher, while ^232^Th and ^226^Ra (0.19 and 0.24) are lower than in this study. The ^226^Ra values reported in Canada and China (Ren et al., 2021) (0.30–0.44) are similar to those in our study. The ^232^Th and ^226^Ra values (0.51 and 0.55) from Zhong et al. (2019) in China are close to our study, while ^137^Cs (1.39) is higher. All CR values in North Kosovo and Metohija (Gulan et al., 2020) are lower (especially ^232^Th and ^226^Ra at 0.1 and 0.09). The highest CR values were reported around the lignite power plant in Western Macedonia (Tsikritzis et al., 2003), with ^137^Cs (43.53) in particular being much higher than the levels measured in this study. These comparisons reveal that the study area generally shows low to moderate levels of radionuclide accumulation.

**Figure 5:**
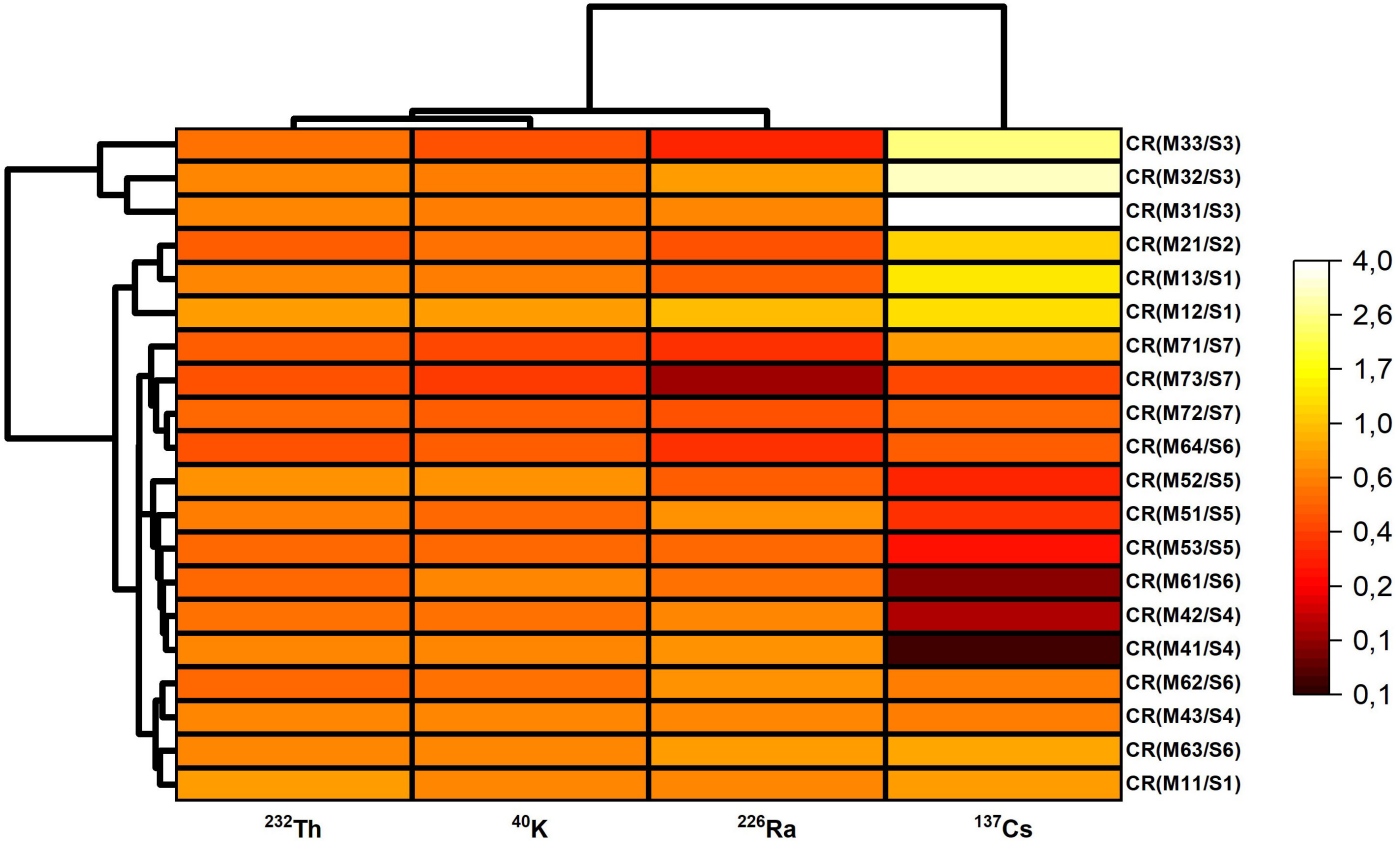
Combined heatmap and dendrogram illustrating the clustering patterns of CR values calculated from the radioactivity concentrations of moss and soil samples.

**Figure 5** heatmap–dendrogram is a visual representation of the CR values ​​in **Table 5** and is in strong agreement with the interpretations of the results obtained. The heatmap-dendrogram shows that the CR values ​​of ^137^Cs are much more variable and higher than those of other radioisotopes in different samples. Some samples show significantly higher bioaccumulation for ^137^Cs, while ^232^Th, ^40^K, and ^226^Ra show more stable and lower bioaccumulation profiles. The dendrogram reveals that similar bioaccumulation patterns naturally cluster, likely reflecting species and/or site effects.

**Figure 6:**
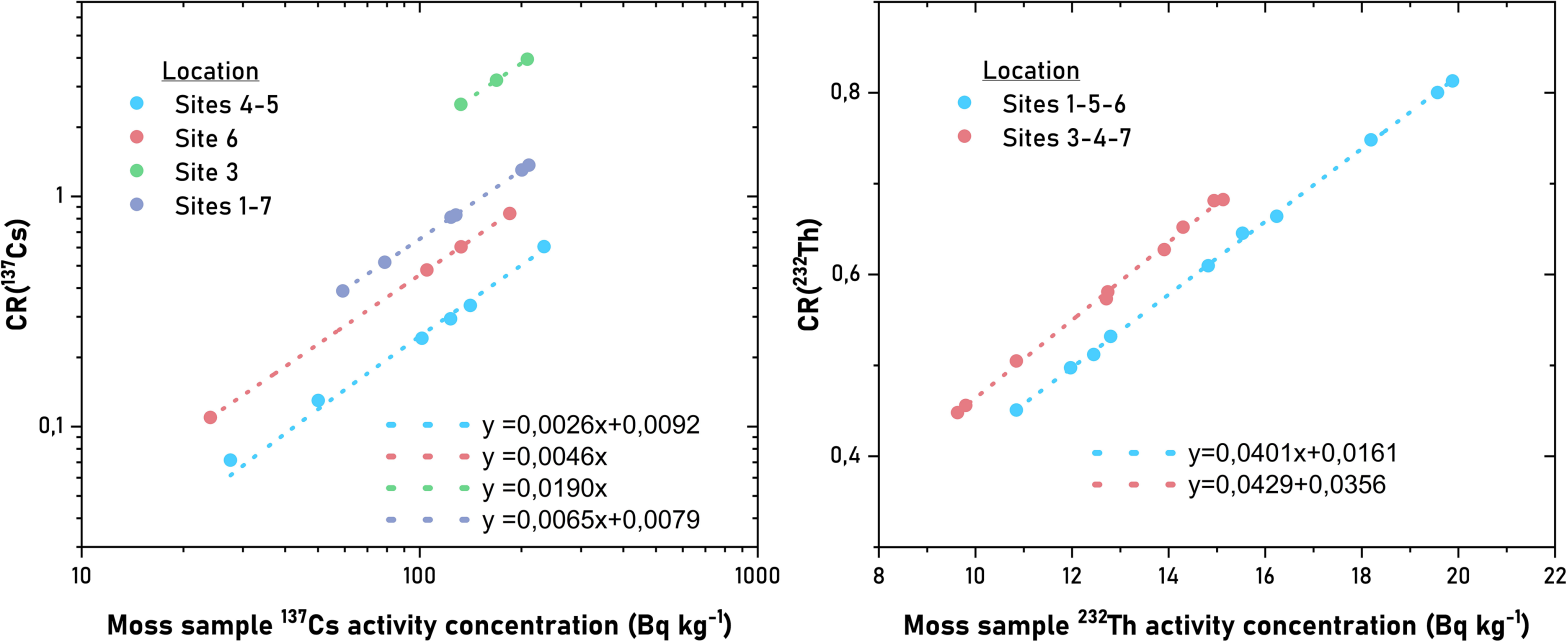
Graphs showing the CR–moss activity concentration relationship for ^137^Cs and ^232^Th.

Comparing the calculated CR values ​​with the activity concentrations of the relevant radioisotopes revealed linear correlations. **Figure 6** presents the CR-activity concentration plots for the ^137^Cs and ^232^Th isotopes. While it provides a clear view of the clustering of samples according to soil activity concentration, it can also provide superficial information about the location and accumulation of moss species.

In both graphs, a steady increase in CR values was observed in parallel with the increase in activity concentration in the moss samples. This situation shows that, unlike the classic transfer relationships in the soil–plant system, the accumulation of radionuclides in mosses is not only dependent on the current level in the soil, but also that the capture and retention processes occurring on the moss surface are enhanced along with the concentration. Particularly for ^137^Cs, distinct linear trends on a logarithmic scale suggest that mosses respond proportionally to rising concentrations of this isotope in the environment (Dowdall et al., 2005). The variation in slope values among different location clusters indicates that CR is sensitive to local conditions even at similar moss activity levels, and that fallout history, soil properties, or microhabitat differences may affect accumulation efficiency.

The relationships obtained for ^232^Th are similarly positive and strong; however, the lower slopes suggest that CR variation is largely determined by a combination of species morphology and local geochemical conditions. Overall, the graphs reveal that moss concentration is not an indicator of passive accumulation but also that CR is a dynamic parameter that changes in a manner sensitive to environmental concentration changes.

### 3.3 PCA analyses

**Figure 7:**
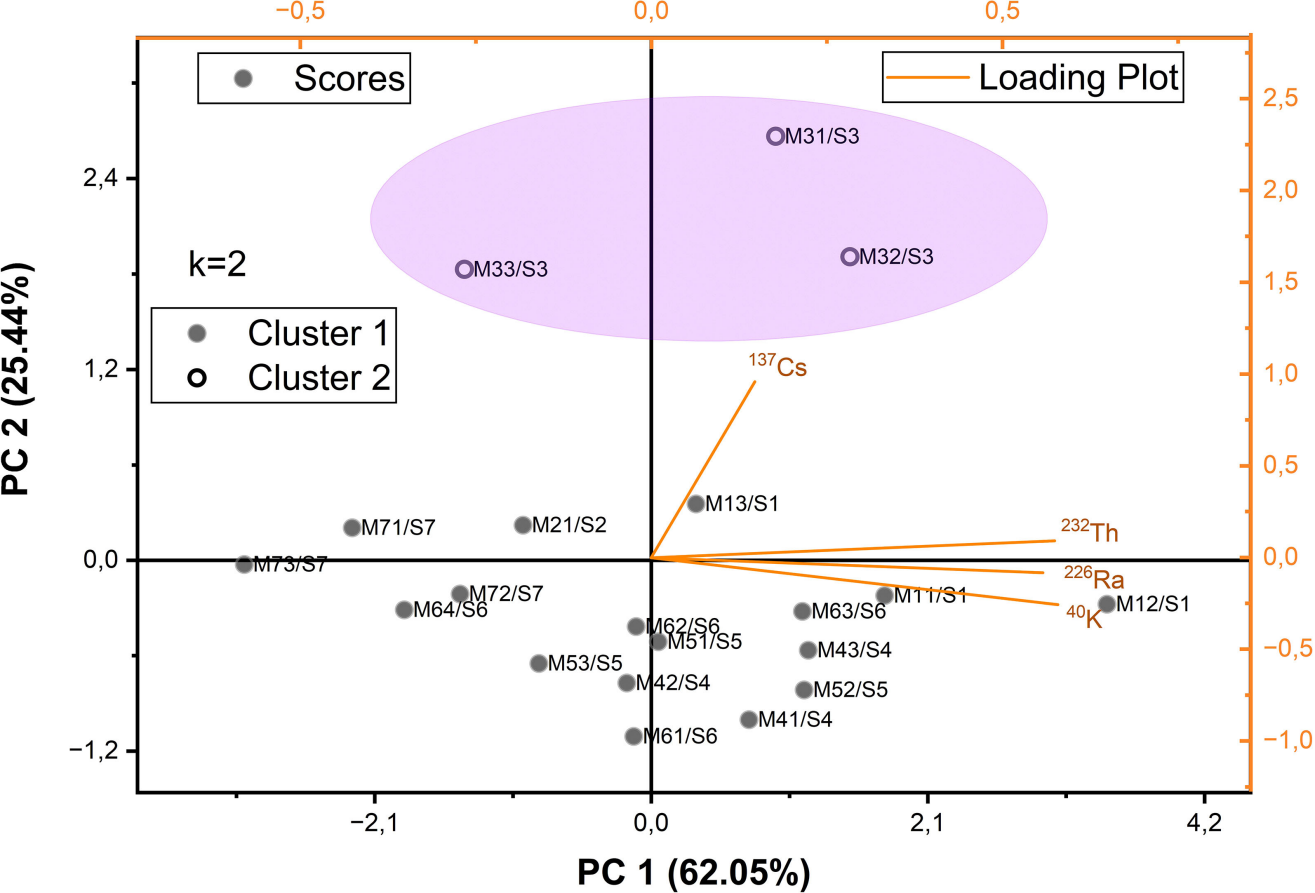
PCA output of CR values ​​and K-Means Cluster Analysis graph.

**Figure 7** presents the results of the PCA performed on CR values together with the corresponding K-means cluster analysis, highlighting similarities and groupings among moss species and sampling sites. PCA results suggest the presence of two dominant environmental variable shaping radionuclide accumulation in mosses. PC1 appears to reflect the influence of natural radionuclides (such as ^232^Th, ^226^Ra and ^40^K), likely representing geogenic control and mineral-bound availability. Samples from Sites 1, 5 and 6 tended to cluster along the positive side of this axis, indicating stronger contributions from substrate-associated activity. In contrast, PC2 seems to be associated with ^137^Cs, pointing toward a depositional gradient that may relate to local fallout history or site-specific microhabitat conditions. Mosses collected from Site 3 showed the highest PC2 scores, consistent with elevated CR (^137^Cs) values and suggesting that this location may have experienced enhanced atmospheric deposition.

Species also exhibited notable structural patterns within the PCA plane. *Grimmia* species generally aligned with higher PC2 values, indicating greater sensitivity to fallout signals, while *Homalothecium* species occupied the lower portions of both axes, possibly reflecting their reduced particle interception capacity. *Syntrichia* species remained in intermediate positions, suggesting a more generalized retention behavior. Overall, PCA highlights that moss communities may record both geogenic and depositional processes simultaneously, with species-specific morphology modulating the magnitude of these signals. *Hypnum cupressiforme* specimens generally exhibit high PC1 and low PC2 values. This suggests that the species exhibits high sensitivity to certain environmental signals but a more limited response in the second dimension of variance, represented by PC2. Its lower-right position in the PCA space emphasizes the specificity of this species’ environmental hoarding and retention behavior to specific conditions.

### 3.4 Evaluation of radiological hazard indices

**Figure 8:**
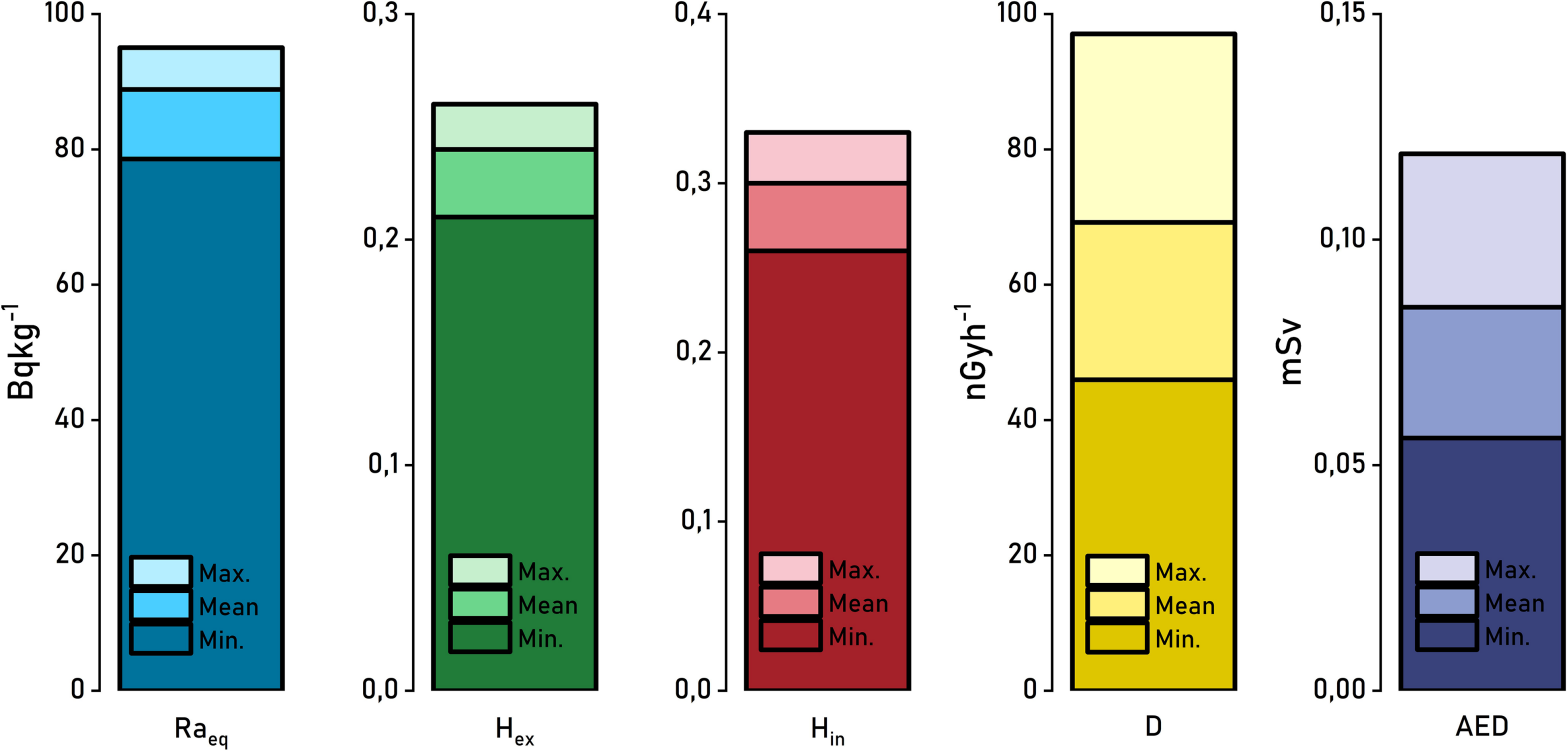
Estimated radiological hazard indices for max, min and mean values of activity concentrations in the soil samples.

Indices grounded in mathematics, such as annual effective doses (AEDE), air absorbed dose rates (D), radium equivalent activity (Ra_eq_), and indoor and outdoor radiation hazard indices (H_in_, H_ex_), are essential for evaluating the radiological risks posed by natural and human-induced nuclides in soil specimens. **Figure 8** shows the radiological hazard indices computed by means of the observed activity concentrations in this investigation. Indices were computed for all samples, with the maximum (max.), minimum (min.), and arithmetic mean values listed.

An essential index for evaluating radiological hazards, Ra_eq_, is calculated for materials containing natural radionuclides. In soil samples, Ra_eq_ varies between 78.58 Bq kg^-1^ and 95.02 Bq kg^-1^, with an arithmetic mean of 88.84 Bq kg^-1^. $Ra_{eq}$ values ​​of soil samples fall below the suggested threshold limit value of 370 Bq kg^-1^ (NEA, 1979) for Radium equivalent activity. H_ex_ values ranged between 0.21 and 0.26, as the H_in_ values varied between 0.26 and 0.33. The arithmetic mean magnitude for H_ex_ and H_in_ in soil subjects were quantified as 0.24 and 0.30, respectively. According to Equations 4 and 5, radiation hazards can be minimized once the H_ex_ and H_in_ magnitudes are less than 1 (Beretka and Mathew, 1985). All the calculated index values for soil subjects in the current research fall well below the established safety threshold, revealing no notable radiological risk.

The outcomes of the gamma radiation rates calculations using Equation 7, excluding the contribution of the ^137^Cs activity level, are as follows: The minimum gamma radiation rates value for soil samples were 37.82 nGy h^-1^, while the highest value was 45.08 nGy h^-1^. The arithmetic mean gamma radiation rates for soil samples were calculated as 42.38 nGy h^-1^. The calculations using Equation 8 revealed lowest gamma dose rates of 45.94 nGy h^-1^, whereas the highest magnitudes were 97.07 nGy h^-1^. The arithmetic mean of activity content in soil samples is 69.20 nGy h^-1^. The evaluation of radiological hazards often involves comparing absorbed dose rates to global standards. The absorbed dose rate averaged across the population, as reported by UNSCEAR (2000), is 59 nGy h^-1^ for outdoor environments and 84 nGy h^-1^ for indoor environments. Excluding the contribution of ^137^Cs activity concentration, the estimates are within acceptable limits. However, the value calculated with ^137^Cs activity concentration for soil samples is above the outdoor world average. These findings highlight the impact of artificial radionuclides on radiological assessments.

According to the projections, outdoor effective doses per year from natural radionuclides in soil subjects vary between 0.046 and 0.055 mSv, with an average of 0.052 mSv. Including ^137^Cs, the magnitudes changed between 0.056 and 0.119 mSv, averaging 0.085 mSv. These annual effective doses are below the global average of 0.07 mSv reported by UNSCEAR (2000). The annual effective dose linked to the overall activity concentration of both natural and artificial radionuclides surpasses the presented value by a small margin.

## 4 Conclusion

-According to the measurement results, the concentration levels of ^40^K, ^226^Ra, ^137^Cs, and ^232^Th in soil subjects were 57.3%, 57.8%, 60%, and 61.2% higher, respectively, than those observed in moss samples. All values ​​except of ^40^K activity level value in the soil subjects were below the world population-weighted averages (420 Bq kg^-1^). ^137^Cs activity concentration was measured above the country average. According to the Shapiro–Wilk test evaluation, activity concentrations are close to a normal distribution.

-The high ^137^Cs activity concentration values ​​reveal the need for monitoring the effects of Chernobyl and reinforce the importance of strategic biomonitoring studies near international nuclear facilities.

-While all hazard indices corresponding to the activity concentrations of natural radionuclides are below the world average, the absorbed gamma dose rate and annual effective dose values corresponding to the total activity concentration for soil samples are slightly above the world average.

-Overall, the contrasting patterns of ^137^Cs and ^232^Th indicate that mosses can reflect several environmental processes occurring at various levels. While ^137^Cs appears to be more sensitive to local atmospheric deposition and microhabitat conditions, ^232^Th seems to reflect the underlying geochemical background. These differences indicate that radionuclide accumulation in mosses is unlikely to be governed by a single mechanism but is instead shaped by a combination of deposition history, surface retention capacity and substrate characteristics.

## Data Availability

The original contributions presented in the study are included in the article/Supplementary Material. Further inquiries can be directed to the corresponding author.
